# Comparison between Electropolymers of 3,5-Dihydroxybenzoic Acid and 2′,6′-Dihydroxyacetophenone in Dimethyl Sulfoxide and Their Analytical Performance towards Selected Analytes with the Role of the Washing Liquid

**DOI:** 10.3390/molecules29163972

**Published:** 2024-08-22

**Authors:** László Kiss, Heng Li, Hui Yan, Sándor Kunsági-Máté

**Affiliations:** 1 Institute of Organic and Medicinal Chemistry, Faculty of Pharmacy, University of Pécs, Honvéd útja 1, H-7624 Pécs, Hungary; kissl@gamma.ttk.pte.hu; 2 János Szentágothai Research Center, Ifjúság útja 20, H-7624 Pécs, Hungary; 3 Fujian Provincial Key Laboratory of Semiconductors and Applications, Collaborative Innovation Center for Optoelectronic Semiconductors and Efficient Devices, Department of Physics, Xiamen University, Xiamen 361005, China; liheng3000@xmu.edu.cn; 4 Jiujiang Research Institute, Xiamen University, Jiujiang 332000, China; 5 Tianjin Key Laboratory of Photoelectric Materials and Devices, School of Materials Science and Engineering, Tianjin University of Technology, Tianjin 300384, China; yanhui@tjut.edu.cn; 6 Key Laboratory of Display Materials and Photoelectric Devices, Tianjin University of Technology, Ministry of Education, Tianjin 300384, China

**Keywords:** 3,5-dihydroxybenzoic acid, 2′,6′-dihydroxyacetophenone, dimethyl sulfoxide, electropolymerization

## Abstract

In the first part of this study, the electrochemical polymerization of two compounds, 3,5-dihydroxybenzoic acid and 2′,6′-dihydroxyacetophenone, was compared in dimethyl sulfoxide solvent on platinum and glassy carbon electrodes. The voltammograms obtained showed remarkable differences between the two monomers and between the two electrode materials. The acetophenone derivative formed electropolymer remnants at the electrodes, while in the case of the benzoic acid derivative, practically no passivation occurred, and the scanning electron microscopic results reinforced this. A few stackings adsorbed only after electropolymerization from a highly concentrated solution of dihydroxybenzoic acid. As a modifying layer on the platinum and glassy carbon electrodes, the prepared films from 2′,6′-dihydroxyacetophenone were tested for tributylamine in acetonitrile and in an aqueous solution of a redox-active compound, hydroquinone, during the stirring of the solution. More stable amperometric current signals could be reached with modified platinum than with glassy carbon, and the significant influence of the organic washing liquid after deposition was established via the study of noise level. In this respect, acetone was the best choice. The amperometric signals with the modified platinum obtained upon the addition of aliquots of the stock solution resulted in a 3.29 μM detection limit.

## 1. Introduction

The electropolymerization of organic molecules has been widely studied, and a number of studies focus on the preparation and characterization of insulating polymers. There are several thousands of organic monomers whose anodic polymerization leads to the growth of such coatings on electrodes. The most typical examples are phenol and its derivatives, but there are a few exceptions where the deposit has good electronic conductivity [1]. The main groups of these compounds are phenols, mesitylenes, and natural phenolic compounds such as tannins, stilbenes, guaiacols, flavonoids, lignans, and phenolic acids, whose electropolymerization results in the blockage of the electrode surface [2]. As a conducting template, the commercially used electrodes include glassy carbon, other carbon-based electrodes, platinum, and gold, which can be used for the investigation of the deposition process and the estimation of the applicability of these coatings as modifiers.

The media of aqueous solutions have gained much popularity for organic layer electrodeposition. In water, the obtained deposits have poor solubility; therefore, it serves as a good medium to obtain the satisfactory yields necessary for coating. In organic solvents, due to the better solvation properties, part of the polymeric product will be removed but it may have favorable structural characteristics. Sensor applications utilize modified electrodes with electrochemically created polymers, mainly because of the improved selectivity and sensitivity [3,4,5,6,7,8]. The prepared modifying layers often have electroactivity themselves as they bear a quinone-type moiety. This is responsible for the observed electrocatalytic property, leading to improved selectivity towards more analytes existing together in the same sample.

Regarding the techniques adopted, potentiodynamic, potentiostatic, and galvanostatic methods are utilized for the preparation of many types (conducting and non-conducting) of deposits. Depending on the desired application, we can determine which method is the best. The most frequently used one is the potentiodynamic category, which covers cyclic voltammetry, where the electrode potential is cycled within a potential range that includes the oxidation or, very rarely, the reduction potential of the monomer. For satisfactory film growth, multiple cycling is applied and a decrease in the peak current with an increasing number of cycles indicates continuous blocking. The change can be very abrupt if the polymer has poor solubility in the solvent and strong adherence. In the potentiostatic category, it is typical for the potential to be set to a constant value appropriated for the charge transfer reaction of the monomer so that it covers chronoamperometry specifically, and film growth can be controlled by fixing the time of electrolysis [9]. The general experience with this mode indicates that the created layer is more compact compared with cyclic voltammetry based on a given electrolysis time. Galvanostatic techniques apply a constant current, but the potential cannot be controlled easily, thus leading to the overoxidation of the product(s). Therefore, this type of method is chosen very rarely. Due to the above-detailed disadvantages of the other techniques, cyclic voltammetry is by far the most powerful, especially when we target a sensor application, as satisfactory porosity is essential for the analyte’s diffusion to the electrode and the product’s diffusion from it.

Generally, when phenols undergo anodic oxidation, the corresponding phenoxyl radical forms and promotes the growth of oligomers or polymers with the other electrogenerated radicals. Whether oligomers or polymers are predominantly formed depends not only on the nature of the monomer but on the solvent and concentration, which also strongly influence this aspect. For example, in a recent work, our group established that para-substituted phenols cannot form coherent deposits in many organic solvents, even at a 50 mM concentration [10].

Hydroxybenzoic acids can readily dissolve in water and in the majority of solvents, but they can be also polymerized through anodic polymerization. The presence of carboxyl group(s) contributes to the increase in the binding sites for ions and molecules through different interactions, thus enhancing the binding abilities. A powerful amperometric sensor was developed for the simultaneous determination of ascorbic acid, dopamine, and uric acid utilizing electropolymerized vanillic acid [11]. The anodic polymer of gallic acid as an electrode modification material made the determination of quercetin possible [12]. The carboxyl group is an electron-withdrawing substituent, so an amino-functionalized hydroxybenzoic acid polymer was synthetized electrochemically with linkages through nitrogen atoms [13].

It is well known that dihydroxybenzenes are electrochemically active and only compounds containing the two hydroxyl groups in the 1,2 and 1,4 positions exhibit reversible redox behavior. Resorcinol and its derivatives, where the hydroxyl groups are in the 1,3 positions, represent a different category as they are highly susceptible to electrode fouling during their electrooxidation. In the first step as in the case of each monomer dimers can form through the formation of carbon–oxygen and carbon–carbon bonds including the dissociation of protons. It was verified both theoretically and experimentally [14,15]. Coherent film growth occurs at all electrodes but there is little difference between electrode materials. The electropolymers composed of resorcinol have practical importance for example in electroanalysis, mainly as molecularly imprinted polymers that are biomimetic recognition materials originating from the electropolymerization of a coating and the analyte molecules coexist with the monomer molecules. Due to the development of recognition sites, a highly selective organic layer makes possible the reliable detection of an analyte as was demonstrated in the work of Hui et al. [16]. They utilized a coating of polyquercetin-polyresorcinol to detect methyl parathion successfully in the presence of similar pesticides with capacitive sensing. Polyresorcinol also proved applicable in capacitive sensing as a molecularly imprinted film towards sulfanilamides in drinking water and milk samples with satisfactory anti-interference properties [17].

Hydroxyacetophenones have a special role in electropolymerization reactions, which also have practical applications. The three isomers of dihydroxyacetophenone were covalently attached to a pyrrole unit and the obtained monomers were electropolymerized through the pyrrole moieties [18]. The acetophenone moieties ensured a highly improved chelating ability towards copper ions and contributed to the electrocatalytic reduction of bromocyclopentane. On the other hand, using the pyrrole modification with the acetophenone moiety ensures the complete separation of the oxidation of pyrrole and the acetophenone moiety as the latter has a relatively high oxidation potential. Thiophene was also modified with dihydroxyacetophenone moieties and electrochemically polymerized through the thiophene units [19]. This thiophene derivative was copolymerized anodically with 3-methylthiophene successfully.

As there are few studies aimed towards the electropolymerization of hydroxyacetophenones, a dihydroxyacetophenone was selected with hydroxyl groups in the 1,3 positions relative to each other. Its electrochemical polymerization was compared with a carboxyl-substituted derivative of resorcinol.

## 2. Results and Discussion

### 2.1. Electrodeposition of the Polymers from Dimethyl Sulfoxide Solvent

In the first part of studies, the electrodeposition process was examined by the two monomers in dimethyl sulfoxide (DMSO), with cyclic voltammetry between 0 and 2 V and a 0.1 V/s scan rate. This solvent was applied due to its favorable properties. However, it is more susceptible to oxidation compared with the other non-aqueous solvents, so this property does not hinder its applicability in preparative electrolyses. No electrode blocking products form in higher concentrations from many monomers by using of this solvent, while electrode deactivation may occur with visible decline of subsequent voltametric peaks in the majority of other solvents. Recently, as a good example, we found that the electrooxidation of the two naphthol isomers results in reproducible voltametric peaks when they are dissolved in 20 mM DMSO [20] while this did not occur in many other organic solvents. Phenol forms a brown deposit from this solvent that is visible with the naked eye and is composed of poly(phenyleneoxide) chains, which has high porosity as earlier studies showed [21].

The number of scans was set to 5 and scans were taken between 0 and 2 V, with a 0.1 V/s scan rate throughout the work, as parameters can highly influence the thickness and structure of coatings. With this number of scans, we can strongly modify the electrolysis time and, consequently, the amount of insulating polymer will highly depend on this. Coverage is a different factor as the polymer can finally be concentrated in stackings, which mainly depends on the solvent.

**Figure 1:**
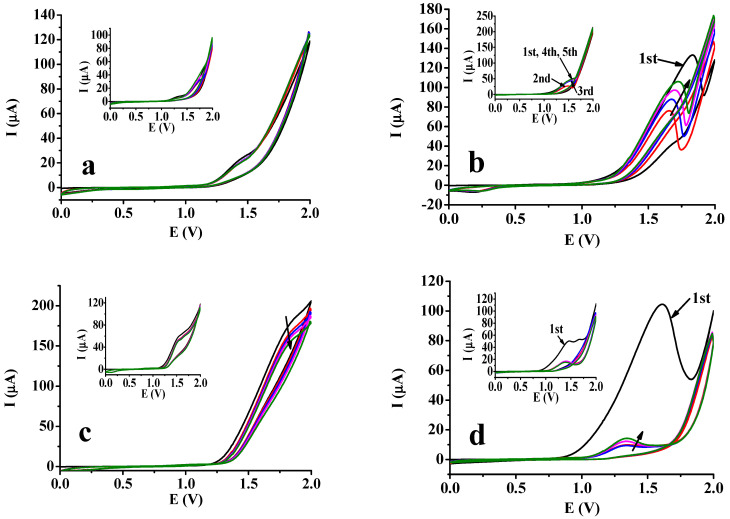
Cyclic voltammograms for 3,5−dihydroxybenzoic acid (**a**) and 2′,6′−dihydroxyacetophenone (**b**) in dimethyl sulfoxide in their 100 mM solutions on a platinum electrode; (**c**) the same for 3,5−dihydroxybenzoic acid and (**d**) for 2′,6′−dihydroxybenzoic acid on a glassy carbon electrode (scan rate 0.1 V/s, supporting electrolyte 100 mM TBAP; the inset graphs show curves recorded in 25 mM solutions and the direction of arrows on curves indicate the direction of the change in voltametric peak heights).

To determine the effect of DMSO on the electropolymerization of 3,5-dihydroxybenzoic acid and 2′,6′-dihydroxyacetophenone, the effect of monomer concentration was examined on platinum and glassy carbon electrodes. Here, 25, 50, 75, and 100 mM were selected as the concentrations. The related voltammograms are shown in Figure 1 for the 25 and 100 mM concentrations. The five voltammograms taken immediately after each other show the differences between the two resorcinol derivatives. Dihydroxybenzoic acid showed no peak but a charge transfer reaction occurs and the shoulder indicates where anodic oxidation takes place in the case of the glassy carbon electrode (at approximately 1.5 V, clearly seen in inset graph of part c by 25 mM concentration). For platinum, a less remarkable sign can be found; consequently, weaker adhesion occurs. Basically, the dihydroxybenzoic acid forms oligomers but its voltametric signal shows large interference with the high background current of the solvent DMSO. The high reproducibility of the voltametric curves verifies that the resulting oligomers were removed from the electrode surfaces.

Studies have been carried out with hydroxybenzoic acids and they state that the electrochemical polymer formation retains the carboxyl groups and there is detailed with the three hydroxybenzoic acid isomers [22]. The units join through ether linkages inside the polymer, as characteristic for phenols, and the authors found that the positions of the linkages were the same starting from the meta and para isomers, leading to the same polymer, and the anodic polymerization of the ortho isomer led to a product where the linkages were in para position relative to each other.

**Figure 2:**
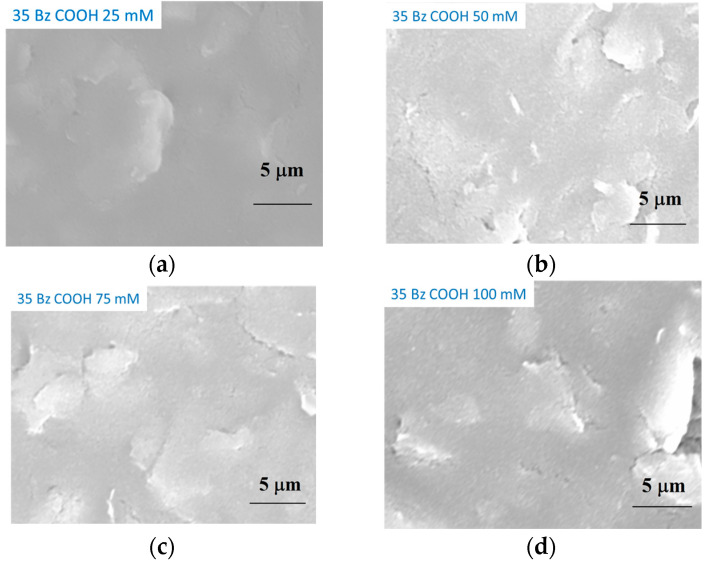

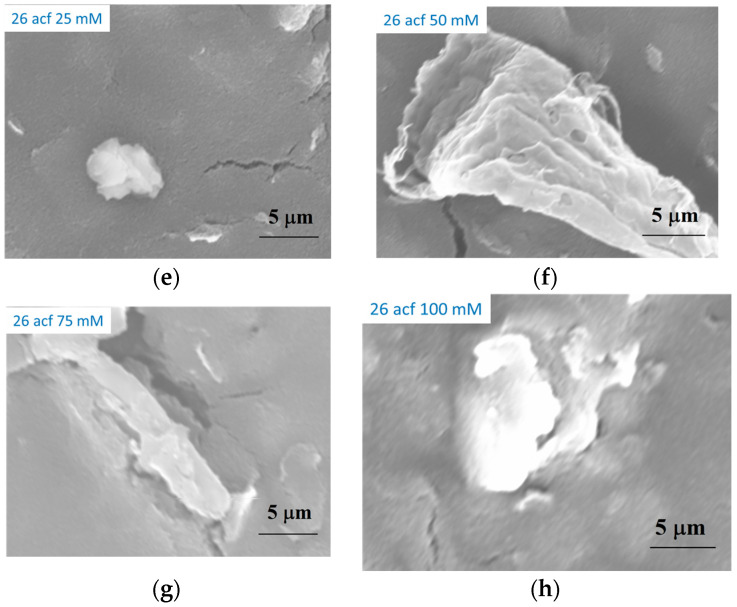
Scanning electron micrographs of electrodeposited films of 3,5-dihydroxybenzoic acid (35 Bz COOH, (**a**–**d**) and 2′,6′-dihydroxyacetophenone (26 acf, (**e**–**h**)) from dimethyl sulfoxide of different concentrations. The second numbers in labels reflect the monomer concentrations: (**a**–**d**) or (**e**–**h**) related to the 25 mM, 50 mM, 75 mM and 100 mM monomer concentrations, respectively.

The dihydroxyacetophenone showed contrarily clear anodic peaks and the decrease in peak height was not monotonic as clearly seen with platinum (Figure 1b). This could be highlighted further with a higher monomer concentration. The obtained voltammograms suggest the development of a coherent deposit, making diffusion through it possible for the molecules. The acetyl group in the benzene ring has a similar effect on the reactions of the resorcinol part as a carboxyl group but the solubility of the polymer is highly influenced by the presence of the methyl group. The elongating polymer chain remains in front of the electrode, making leading to longer molecular weight chains, and the aromatic interactions between them ensure coherency between them. Together with the previous findings, it can be concluded that the –OH group in the dihydroxybenzoic acid ensures favorable solvation properties. This effect is significantly stronger in the case of a glassy carbon electrode, probably due to the strong apolar interactions with the aromatic carbon chains packed densely in the bulk material of the electrode. In the 25 mM solution, there were two peaks, indicating that there is a film rupture under development in the first scan. At higher concentrations, only one peak is found, showing that the rate of film propagation at the surface of the electrode is enhanced, hindering the rupture process (Figure 2).

The micrographs clearly show that there are remarkable differences between the deposits of the two monomers. On the uneven surface of the carbon electrode template, there were no stackings in the case of 3,5-dihydroxybenzoic acid but at the highest 100 mM concentration, in accordance with the voltametric results, as products mainly dissolved. In contrast, when 2′,6′-dihydroxyacetophenone was anodically polymerized, stackings were observed at all concentrations, verifying earlier observations.

### 2.2. The Electrooxidation of the Two Monomers in DMSO–Acetonitrile Mixtures

The results discussed in the previous section highlighted that the deposit formed from 3,5-dihydroxybenzoic acid does not remain on the surface of the platinum electrode, so a co-solvent was needed in the investigations. As experienced in some earlier studies, the polymers prepared with electropolymerization in acetonitrile are generally scarcely soluble in this solvent, so we tried to utilize the advantages of the two organic solvents. Therefore, solution series were prepared by varying the volume fraction of acetonitrile, 25, 50, 75 and 100 *v*/*v*%, respectively, and the concentrations of monomers were also 25, 50, 75, and 100 mM for each solvent composition, respectively, as in the previous experiments. The pure acetonitrile acted as a control solvent to make the assessment of the effect of DMSO easier.

**Figure 3:**
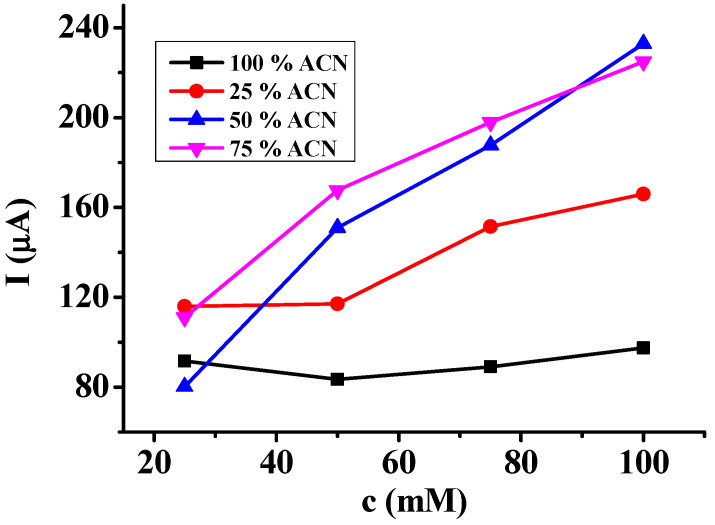
The dependence of the first peak currents on the concentration of 3,5-dihydroxybenzoic acid by varying the acetonitrile (ACN) content in *v*/*v* %.

Figure 3 summarizes the results in different solvent mixtures by showing the dependence of the heights of the first voltametric peaks on the concentration. As can be expected in pure acetonitrile, there were no differences between the four concentrations, so the studied concentrations were too high to find any tendency. In other words, the rate of surface blocking showed no variation during the timescale of the first scan.

In contrast, remarkable dependence could be observed in the DMSO–acetonitrile mixtures and the magnitudes were significantly higher compared to any concentration. The viscosity of DMSO is approximately 4-fold higher than that of acetonitrile and taking into account this fact, the current enhancements are reasonable—elevating the acetonitrile content to increase the diffusion coefficient of monomer. The dependence of peak heights on the concentration can be attributed to the identical solvation properties of DMSO as it highly contributes to the formation of layers with high porosities. As pure acetonitrile has the lowest viscosity of all the studied liquids, the fact that the currents were the lowest can only be explained by the development of a compact deposit. This also verifies the poor solvation properties of acetonitrile for polymer of 3,5-dihydroxybenzoic acid. On elevating its content in the mixtures, there were more peaks and sharper peaks, providing an additional indication for stronger adherence by remarkably excluding the DMSO molecules, thus obviously contributing less to the background currents. 

### 2.3. Assessment of the Electrodeposited Films in the Electrooxidation of Tributylamine

**Figure 4:**
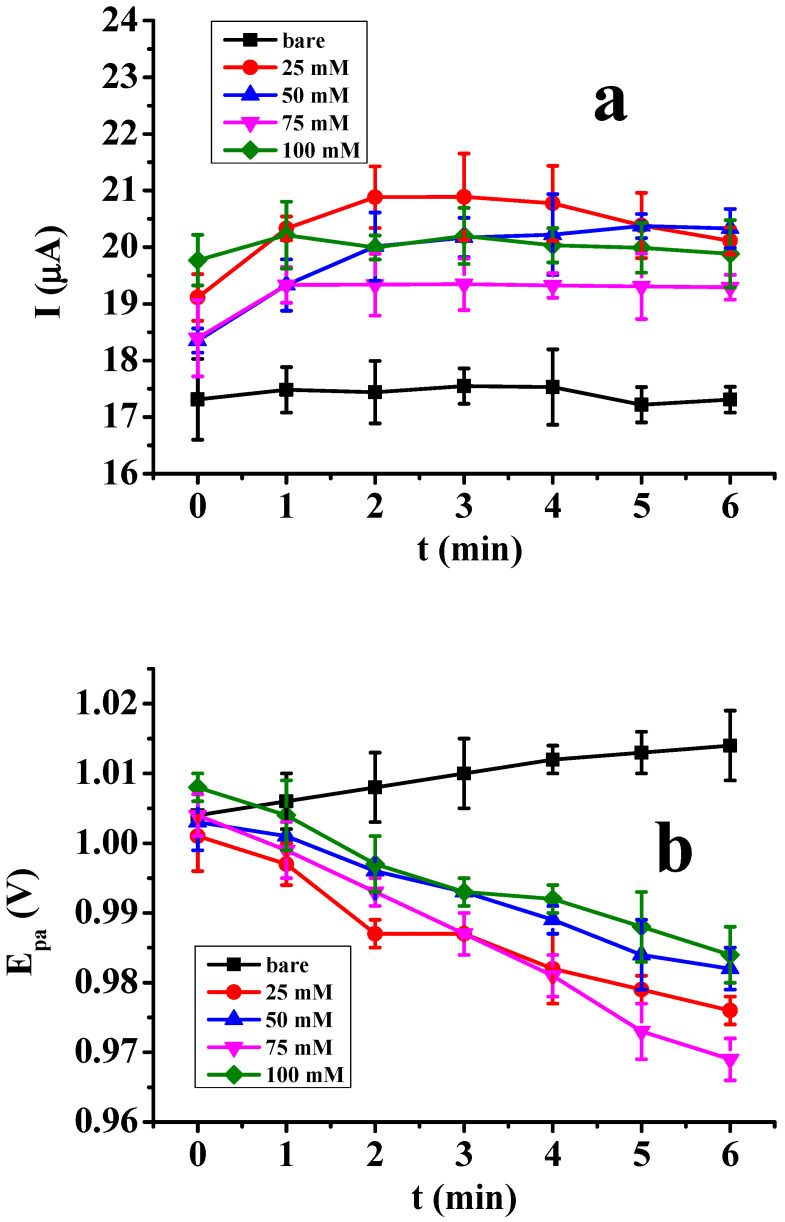
The dependence of peak currents (**a**) and peak potentials (**b**) on the immersion time of poly(2′,6′-dihydroxyacetophenone) in 10 mM acetonitrile solution of tributylamine containing 20 mM TBAP.

The compound tributylamine is interesting as an intermedier in organic syntheses and it can serve as an appropriate model compound for binding studies. So, this was one of the analytes studied here as it has an amino group ensuring bonding to electrodeposited layers as well as enabling atoms to bind molecules through hydrogen bonds. The performance of poly(2′,6′-dihydroxyacetophenone) using platinum as a template can be seen from the results displayed in Figure 4, with averages of three parallel measurements. The peak currents increased independently on the concentration of monomer compared with the bare electrode, suggesting the binding abilities of layers to tributylamine through hydrogen bonds, leading to some degree of accumulation. The curves have a current maximum, which shows that the structure of deposits changed during exposition of the solvent. On the other hand, the peak potentials continuously decreased in the case of modified electrodes by each concentration in time in contrast to the unmodified electrode. This is also in accordance with the effect of adsorption close to the electrode surface. Signal enhancement due to a deposit seems to be very promising itself but showed instability in time. If the dependence on time would lead to a saturation curve. this could guarantee the reliability of the modified electrode for further analytical studies. These findings show that the poly(2′,6′-dihydroxyacetophenone)-modified electrode cannot be considered in the determination procedure for tributylamine in non-aqueous conditions.

### 2.4. The Effect of Electrode Material on Response to Stirring

In many electroanalytical experiments carried out in aqueous solutions, dihydroxybenzaldehydes bearing a *o*-quinone moiety are used to enhance the electrocatalytic activity of carbon-based electrodes. In particular, 3,4-dihydroxybenzaldehyde is popular in this respect in its electropolymerized form due to its scarce solubility in water. For example, its electrocatalytic property could be utilized for oxidative detection of ***β***-nicotinamide adenine dinucleotide [23] and similar work also made possible the simultaneous detection of formate ions and glucose-6-phosphate with such dinucleotides [24]. Electrode modification with this benzaldehyde derivative led to a decrease in the overpotential of ascorbic acid anodic oxidation so its simultaneous quantification was possible in the presence of uric acid [25]. However, these molecules also had electrochemically active quinone parts after electrodeposition, ensuring powerful properties utilized in electroanalysis. The polymers investigated herein may have interesting properties, so they were studied for similar redox-active compounds as earlier in the literature.

**Figure 5:**
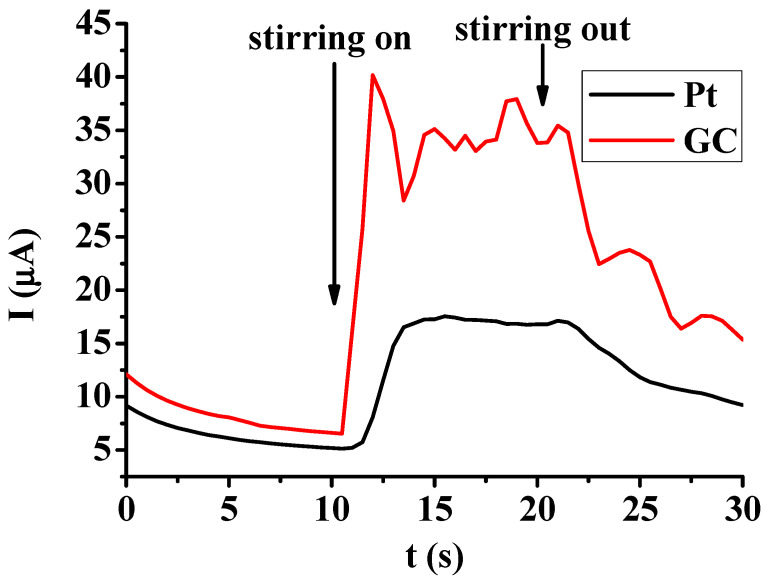
Chronoamperometric curves of glassy carbon and platinum electrodes modified with poly(2′,6′-dihydroxyacetophenone) after washing with acetone with response to stirring at 700 rpm in 5 mM solution of hydroquinone with pH set to 7 with 0.05 mol/L phosphate buffer.

The effect of electrode pretreatment was also investigated in a aqueous solution of 5 mM hydroquinone buffered to pH = 7 with 0.05 mol/L phosphate buffer. The modified electrodes with poly(2′,6′-dihydroxyacetophenone) exhibited approximately 0.15 V smaller peak potentials than the bare electrodes. A similar change was observed when five cycles were taken in pure DMSO, highlighting that the solvent has a predominant effect on the electrodes during the pretreatments. Generally in amperometric methods, stirring is applied to enhance the mass transport of analyte to the electrode and therefore the sensitivity while the electrode is polarized to a potential, which allows a diffusion-controlled heterogeneous process. The organic layers of poly(2′,6′-dihydroxyacetophenone) deposited onto platinum and glassy carbon are compared in Figure 5 in response to stirring in the 5 mM solution of hydroquinone by applying a 700 rpm stirring speed and a 0.5 V constant potential. The choice of this potential seems to be optimal as the electrooxidation of hydroquinone has a diffusion-controlled nature, which was checked previously with cyclic voltammetry. There are remarkable differences between the two electrodes and the curves provide additional information about the coherence of electrodeposited layers (Figure 5). In the case of the glassy carbon electrode, the change was higher and the response was faster towards the start of stirring. In contrast, the modified platinum electrode showed a smaller change but the curve has a smoother horizontal line during stirring and the response was slower. These observations allowed contradictory conclusions regarding the coherence of films. Previously in the electrodeposition experiments, a glassy carbon electrode showed a higher deactivation speed during electropolymer formation from 2′,6′-dihydroxyacetophenone, but a relatively free diffusion takes place in the stirring experiment, further verified through the clearly visible noise of stirring, which is rather characteristic for the unmodified electrodes.

Polymeric films prone to ageing processes can be accelerated with an appropriate solvent through enhancing segment–segment interactions. As after the electrodeposition step, the removal of unreacted monomers, supporting the electrolyte and the solvent, is required for further experiments, the electrode should be washed with a pure solvent that evaporates rapidly. During washing, the solvent used can modify the structure of organic deposit, thus choosing an optimal solvent demands additional investigation. The same amperometric procedure seemed to be the best for the estimation of layers as the strength of the stirring noise can provide very useful information about the coherencies of layers. More non-aqueous solvents were tested in this respect through the electrode response towards stirring with the platinum electrode. The best indicator for it is the study of the noise level which means the average magnitude of the current oscillation. Of the studied solvents, dimethyl sulfoxide was also present also used in the electrolysis solution and due to its relatively low volatility, it was removed from the electrode with careful removal by using laboratory tissue paper and the residual of the solvent was finally left to dry. In the case of other solvents, a few times was enough for complete evaporation. This noise level ranged between 5 and 10 μA for most solvents (dimethyl sulfoxide, dimethyl formamide, dichloromethane, acetic acid, tetrahydrofuran), and between 2 and 3 μA for ethanol and ethyl acetate, 1.3 μA for acetonitrile and 0.4 μA for acetone. As shown in the data, acetone seemed to be the most optimal for washing, leading to a coherent film which can withstand stirring while the current response is significantly higher than in the quiescent solution. This also indicates that fast diffusion of the analyte occurs through the organic deposit. On the other hand, these results suggest that it is advantageous in some cases if more solvents are applied consecutively during the preparation of films. Here, DMSO made possible the formation of a significant amount of polymeric material with a structure that can then be modified properly. The use of acetone as a solvent during the deposition and for washing would not elevate this, as a low amount of polymer will grow on the surface of platinum. Apart from minimization of the disturbance caused by stirring, ensuring curve smoothing is not required, the deposit formed during washing with acetone elevated the diffusion of analyte molecules.

**Figure 6:**
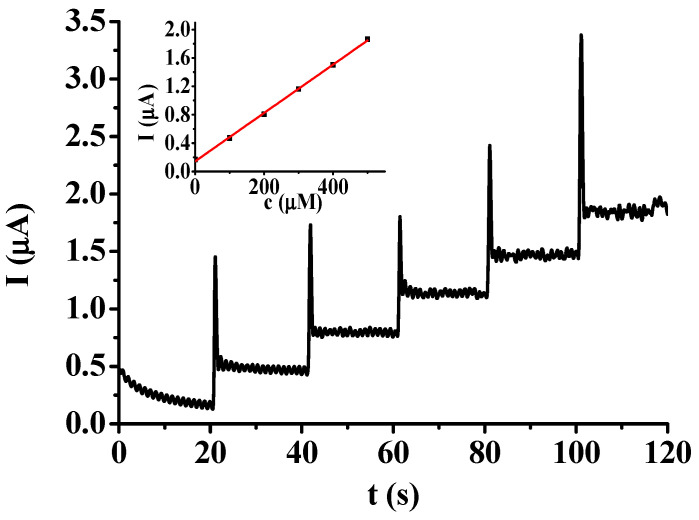
Amperometric curve for hydroquinone during stirring at 700 rpm by sequential addition of stock solution for concentrations 0, 100, 200, 300, 400, and 500 mM with a poly(2′,6′-dihydroxyacetophenone)-modified platinum electrode (supporting electrolyte 0.05 M pH = 7 phosphate buffer, constant potential 0.5 V).

From an analytical perspective, the stability of the signal plays a more important role than its magnitude in spite of the fact that analytical improvements focus on the increase in signal; on the other hand, the background noise highly influences detectability. Therefore, the use of the modified platinum electrode seemed to be better for electroanalytical experiments, washed with acetone after electrodeposition. The calibration curve was determined by applying a 700 rpm stirring speed on adding the necessary aliquots of the stock solution of hydroquinone to 0.05 mol/L pH = 7 phosphate buffer and by setting 0.5 V as the constant potential. The additions were repeated in every twentieth second (Figure 6). Obviously, after each addition, a delay could be seen in accordance with the previous findings due to the coherence of the organic film.

The inset graph illustrates the dependence of currents after the stock solution additions on the concentration of hydroquinone and a straight line indicates linear dependence. The equation of this curve was *I*(μA) = 0.142(±0.014) + 0.00343(±0.00004)*c*(μM) by taking the average of three calibrations. To use the 3σ criterion for the establishment of the detection limit, three measurements were carried out in stirred conditions in 0.05 M phosphate buffer and scattering was calculated. As a result, 3.29 μM was the detection limit.

### 2.5. The Signal Amplification Ability of Poly(2′,6′-Dihydroxyacetophenone) for Selected Compounds

As organic solvents can easily penetrate through the organic deposited layers, the effect of the porosity and degree of accumulation can be studied with an analyte. More redox-active organic compounds were selected by dissolving them in acetonitrile in a 5 mM concentration for both platinum and glassy carbon electrodes and the electrodeposited poly(2′,6′-dihydroxyacetophenone) layers were estimated. These materials were all aromatic (hydroquinone, 2,5-dihydroxybenzoic acid, 2′,5′-dihydroxyacetophenone, 4-methoxyphenol) with or without weak susceptibility to polymerization and their peak heights were compared with bare and modified electrodes, as this is an easy procedure to gain useful information about the deposit. The peak current recoveries ranged between approximately 85 and 100% for each compound (Table 1) on both platinum and glassy carbon. In spite of these data, significant accumulation can be recognized as they were close to 100% and the porosity alone reduces the mass transport to a greater extent when a deposit covers the active surface coherently.

## 3. Materials and Methods

The chemicals used were at least of analytical reagent grade, each investigation was carried out in organic solvents, tetrabutylammonium perchlorate (TBAP) was used as a supporting electrolyte, and in aqueous solutions, pH = 7 was set with 0.05 M phosphate buffer, which elevated solution conductivity. The platinum and glassy carbon discs as working electrodes had were of 1 mm diameter. These electrodes were sealed in polyetheretherketone as an insulating sheath (eDAQ, Sydney, Deniston East, Australia). To build up the three electrode cells, in addition to the working electrodes, a platinum–iridium rod was applied as a counter and a silver wire as a reference electrode in organic media. A standard calomel electrode served as a reference in aqueous solvents. The three-electrode cells were connected to a potentiostat (Dropsens, Oviedo, Spain). The electropolymerization experiments were carried out according to the usual protocol by the cyclic voltammetry method (running a series of voltammograms) and finally the obtained deposits were washed to remove the unreacted monomers and supporting electrolyte with the corresponding solvent.

Before all voltametric experiments, a polishing procedure was applied where a polishing cloth was immersed in the aqueous suspension of alumina powder with 1 μm average diameter. In this way, the blocking materials originating from the electropolymerization processes were removed and then thoroughly washed with tap water and finally with distilled water. After the preparation of organic layers, the electrodes were thoroughly washed with dry acetone and this procedure ensured the complete removal of soluted materials and solvent used for electrolysis.

For the visualization of the morphology of the deposited layers, a JEOL JSM-6300 scanning electron microscope (Osaka, Japan) was used. This equipment used a 20 kV acceleration voltage.

## 4. Conclusions

The investigations showed that only the deposits grown from 2′,6′-dihydroxyacetophenone might gain practical application when the solvent dimethyl sulfoxide is used in the electrolyzing solution. In light of the results, it can be supposed that 3,5-dihydroxybenzoic acid might be used as a copolymer with scarce solubility. Planning of each step during deposition may be critical as the nature of the washing solvent highly influences the properties of the coverage, highlighting the importance of the final washing step at the end of preparation.

## Figures and Tables

**Table 1:** Peak current recoveries in % for poly(2′,6′-dihydroxyacetophenone)-modified electrodes.

Compound	Pt Electrode	Glassy Carbon Electrode
4-methoxyphenol	90.4	92.7
Hydroquinone	90.5	86.8
2,5-dihydroxybenzoic acid	91.5	89.2
2,5-dihydroxyacetophenone	96.5	89.6

## Data Availability

The data presented in this study are available on request from the corresponding author.
